# De Novo RB1 Germline Variant in Retinoblastoma with Two Subsequent Independent Neoplasms: Case Report and Literature Review

**DOI:** 10.3390/ijms252212338

**Published:** 2024-11-17

**Authors:** José de Jesús Pérez-Becerra, Víctor Ulises Rodríguez-Machuca, María Teresa Alejandra González-Rodríguez, Sinhue Alejandro Brukman-Jiménez, Alfredo Corona-Rivera, Juan Antonio Ramirez-Corona, Idalid Cuero-Quezada, Jorge Román Corona-Rivera, Xóchitl Aurora Ramírez-Urenda, Graciela González-Pérez, Felipe de Jesús Bustos-Rodríguez, Lucina Bobadilla-Morales

**Affiliations:** 1 Human Genetics PhD Program, Department of Molecular Biology and Genomics, Centro Universitario de Ciencias de la Salud, Universidad de Guadalajara, Guadalajara 44340, Mexico; perezbecerra95@gmail.com (J.d.J.P.-B.); vu.rodriguez11@gmail.com (V.U.R.-M.); maria.gonzalez0181@alumnos.udg.mx (M.T.A.G.-R.); jar.corona93@gmail.com (J.A.R.-C.); idalidcuero95@gmail.com (I.C.-Q.); 2 Human Genetics Institute “Dr. Enrique Corona Rivera”, Department of Molecular Biology and Genomics, Centro Universitario de Ciencias de la Salud, Universidad de Guadalajara, Guadalajara 44340, Mexico; alcoronar@gmail.com (A.C.-R.); rocoronar@gmail.com (J.R.C.-R.); 3 Cytogenetics Unit, Hospital Civil de Guadalajara Dr. Juan I. Menchaca, Guadalajara 44340, Mexico; brukman.alejandro@gmail.com; 4 Pediatric Hematology and Oncology Department, Hospital Civil de Guadalajara Dr. Juan I. Menchaca, Guadalajara 44340, Mexico; xaramirez@hcg.gob.mx; 5 Ophthamology Department, Hospital Civil de Guadalajara Fray Antonio Alcalde, Guadalajara 44280, Mexico; 6 Department of Anatomy and Pathology, Hospital Civil de Guadalajara Dr. Juan I. Menchaca, Guadalajara 44340, Mexico; fbustos@hcg.gob.mx

**Keywords:** retinoblastoma, *RB1*, subsequent neoplasms, *RB1* germline pathogenic variant, de novo variant

## Abstract

Variants in the *RB1* gene are associated with retinoblastoma (RB) development, and their presence in germline cells considerably increases the risk of subsequent malignant neoplasms (SMNs) in RB survivors. We report a female patient with bilateral RB who developed two SMNs in less than ten years, with a de novo pathogenic nonsense variant in *RB1* [NM_000321.3:c.306T>A, p.(Cys102*)] in heterozygosity. The updated literature review of similar cases of SMN in patients with a previous diagnosis of RB reveals a wide range in both the type of subsequent malignancy and the age at which these SMNs develop. In addition, we identified only three cases with two SMNs following RB diagnosis, with at least one of these being an EWS. This case broadens the clinical and genetic landscape of RB, demonstrates the importance of a multidisciplinary approach in these patients, and highlights genetic diagnosis as a mandatory feature for management.

## 1. Introduction

Retinoblastoma (RB) is a rare childhood cancer. However, it accounts for most ocular neoplasms that develop in pediatric patients, with an overall incidence of 1 in 15–20,000 live births. RB can be divided into two major groups: hereditary and non-hereditary. The hereditary group accounts for about 30% of all cases, with a mean age at diagnosis of 12.3 months, usually with bilateral disease, and is characterized by germline cytogenomic alterations in the *RB1* gene [1]. Being a tumor-suppressor gene, *RB1* germline inactivation in patients previously diagnosed with hereditary RB has up to 20 times higher susceptibility to develop subsequent malignant neoplasms (standardized incidence ratio (SIR) = 20.4 (confidence interval (CI) 15.6–26.1)) (SMN) [2,3,4,5,6], and the risk of presenting a third neoplasm after a second is up to 8 times higher compared to the general population risk (SIR = 8.5 (CI 3.7–16.7)) [7,8]. In clinical practice, standard RB treatment with radiotherapy and chemotherapy (especially when using alkylating agents) has been associated with an increased risk of developing SMNs [9]. A wide variety of SMNs have been reported in RB survivors, such as leukemias and lymphomas (4%), melanoma (8%), carcinomas (13%), central nervous system tumors (3%) and sarcomas (60%), the latter being the most common among patients with RB. Ewing sarcomas (EWSs) accounts for less than 5% of all SMNs in patients with a previous diagnosis of RB [9,10,11]. Herein, we aim to present a patient with RB who presented two SMNs associated with a germline pathogenic variant in *RB1* detected by Next Generation Sequencing (NGS).

## 2. Case Presentation

**Figure 1:**
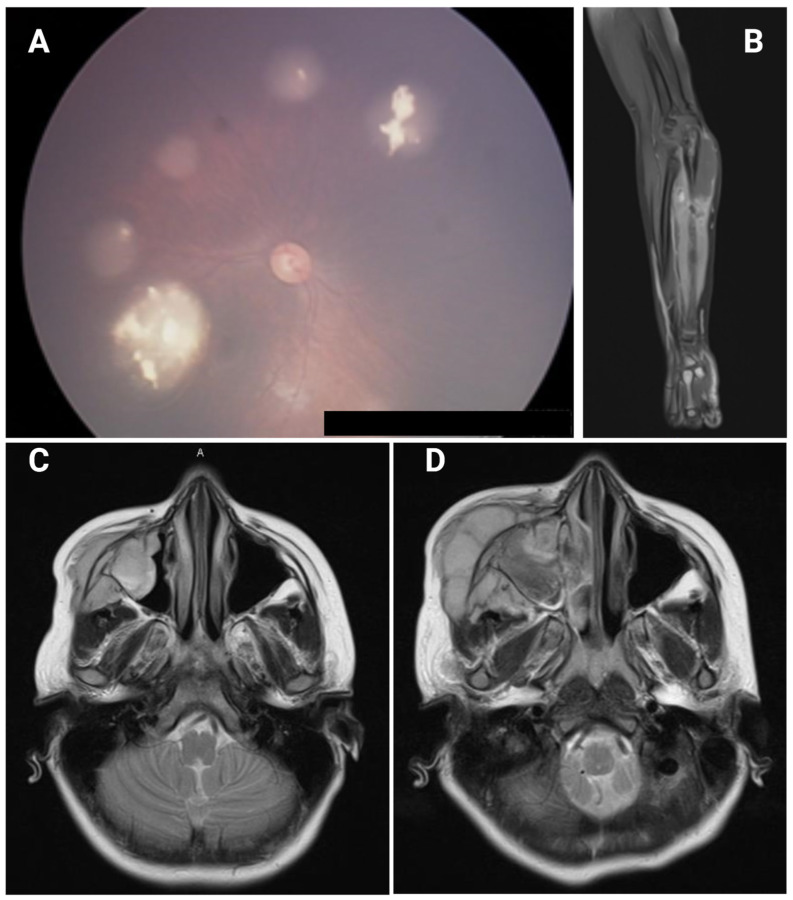
(**A**) Multifocal RB in the right eye with calcium content in its interior. (**B**) MRI of the left forearm showing a hyperintense lesion in the cubitus with disruption of bone cortex. (**C**) Head MRI at time of diagnosis showing a giant lobulated tumor in the right maxilla with extension to the ipsilateral maxillary sinus space. (**D**) Head MRI three months after diagnosis showing a giant lobulated tumor in the right maxilla with extension to the ipsilateral maxillary sinus space.

The proband, a female infant, presented with bilateral leukocoria at three months of age and was referred to the pediatric ophthalmology service, where bilateral RB without optic nerve infiltration was diagnosed (Figure 1A). The tumor in the left eye was classified as group E according to the International Classification of Retinoblastoma (ICRB) and was enucleated; the tumor in the right eye was classified as group B and received transpupillary thermotherapy (TTT). No evidence of optic nerve infiltration was found as assessed by magnetic resonance imaging (MRI) or infiltration at bone marrow by biopsy. Adjuvant chemotherapy was administered in six cycles of VCE (vincristine, carboplatin, etoposide) and resulted in complete remission. The patient was monitored with annual follow-up appointments with hemato-oncology. We conducted a comprehensive evaluation in the genetics department, with no hereditary history of cancer or relevant pathological background, and initiated a cytogenomic approach in search of germline *RB1* gene variants.

The first SMN was diagnosed at five years of age when she presented with pain and increased volume in the left forearm due to a cubital tumor (Figure 1B). A biopsy was performed, and the histopathologic result showed small round blue cell neoplasia with bone infiltration compatible with EWSs. She was treated with a supracondylar amputation of the left forearm, followed by adjuvant chemotherapy, with a complete post-treatment response observed and no evidence of residual neoplasia.

The second SMN presented at the age of 9 years, with the patient reporting pain and an increase in volume in the right maxillary region. An MRI showed a lobulated tumor in the right maxilla and maxillary process of the zygomatic bone with extension to the ipsilateral maxillary sinus space (Figure 1C,D). Biopsy results indicated a sarcoma with CD99-positive immunohistochemistry. Due to the aggressive behavior of this neoplasm and the compromise of facial structures, palliative care was offered with metronomic chemotherapy and pain management. The patient evolved torpidly, and three months after diagnosis, she died due to complications of the third neoplasm.

**Figure 2:**
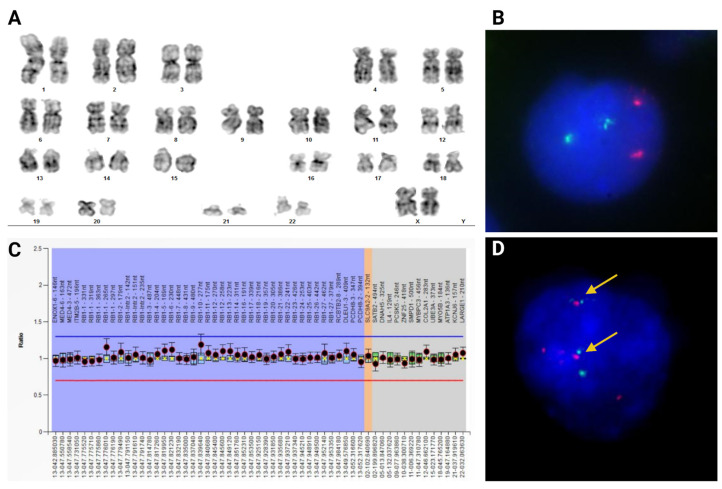
(**A**) Karyotype of the patient with G-band staining showing a 46,XX[20] result. (**B**) FISH of interphase chromosomes on peripheral blood cells showing a nuc ish (*RB1*,*LAMP1*)x2 [200] result. (**C**) MLPA copy number variant analysis of *RB1* gene showing an rsa 13q14.2(*RB1*)x2 result. (**D**) FISH of interphase chromosomes of third neoplasm (Ewing sarcoma) showing fusion between *EWSR1*::*ERG* genes (yellow arrows).

**Figure 3:**
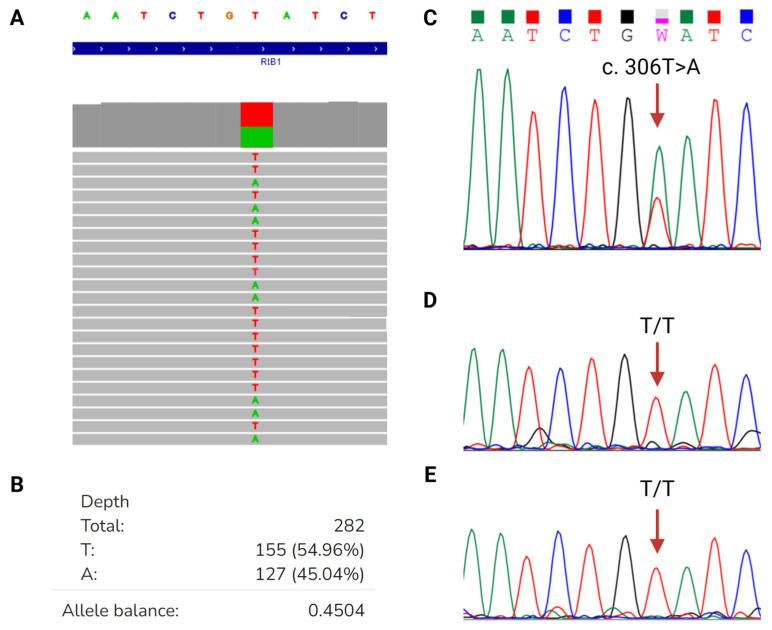
NGS analysis and Sanger sequencing *trio* analysis of the identified variant in our patient. (**A**) NGS analysis of *RB1* gene in the patient. (**B**) Depth of reads and variant allelic balance in the patient’s *RB1* gene. (**C**) Sanger sequencing of the patient showing a transversion (T>A) in nucleotide 306 of the coding DNA. (**D**) Electropherogram of the father. (**E**) Electropherogram of the mother.

Karyotype analysis results were 46,XX (Figure 2A); fluorescence in situ hybridization (FISH) and Multiplex Ligation-dependent Probe Amplification (MLPA) analysis revealed two copies of the *RB1* gene (Figure 2B,C). Through NGS analysis, a heterozygous NM_000321.3 (RB1):c.306T>A, (p.Cys102*) variant was identified in our patient (Figure 3). The *RB1* gene of both parents was normal, although paternal identity was assumed but not confirmed. FISH analysis of tumor tissue from the second SMN revealed the *EWSR1/ERG* fusion gene (Figure 2D).

## 3. Discussion

The patient presented in this case report with early-onset bilateral RB developed two independent SMNs due to a germline de novo pathogenic variant in *RB1*. We identified a NM_000321.3:c.306T>A, p.(Cys102*) nonsense *RB1* pathogenic variant, which was classified as pathogenic according to American College of Medical Genetics (ACMG) variant classification guidelines [12], based on: (a) its effect at the protein level since it generates a null allele in a gene whose loss of function is a known mechanism of disease, (b) its low frequency based on gnomeAD population databases (<0.05%) and (c) de novo data, regarding a patient with consistent phenotype (d) reputable source data, reputable source recently reports variant as pathogenic; *RB1* c.306T>A, nonsense variant classified as Pathogenic [13].

Exposure to radiotherapy and the administration of specific chemotherapeutic agents elevate the risk of SMN, especially in patients with hereditary RB [14]. A threefold increase in SMN occurrence in patients with hereditary RB treated with radiotherapy compared to patients with non-irradiated hereditary RB was found in a U.S. [3] and a Dutch cohort [4]. Exposure to radiotherapy also affects the onset of SMNs, as patients with hereditary RB treated with radiotherapy present early-onset SMNs more frequently than patients with non-irradiated hereditary RB [11]. There appears to be a dose-response relationship between high doses of radiotherapy and a higher risk of developing osteosarcoma and soft tissue sarcoma [15]. Treatment with anthracyclines and alkylating agents is another known risk factor for the development of SMNs in cancer survivors [16]. In a U.S. cohort of patients previously diagnosed with RB, systemic chemotherapy was identified as a risk factor for SMNs [3]. An excess in leukemia incidence in RB survivors treated with eye-preserving chemotherapy was identified in Argentine [17] and German [18] cohorts. Chemotherapy regimens that include alkylating agents and topoisomerase inhibitors are the most used in RB, and their possible involvement with the presence of subsequent tumors is known, especially when combined with radiotherapy [19]. However, the impact of chemotherapy without radiotherapy in patients with hereditary RB is poorly described [18].

Environmental factors play a fundamental role in developing multiple types of cancer. Generally speaking, these factors include air pollution, exposure to chemicals and radiation [20]. In the particular case of retinoblastoma, because of its embryonic origin, the environmental risk factors will be those to which the parents were exposed before or during pregnancy. Smoking, exposure to pesticides, toxic air and residence near industrial areas have been linked to the development of retinoblastoma [20,21,22]. For Ewing’s sarcoma, the environmental risk factors will also include parental occupational exposures, including but not limited to exposure to herbicides, insecticides and fertilizers [23]. The patient’s parents deny exposure to any known environmental risk factors from occupational exposures or tobacco use before or during pregnancy, and the patient herself was not exposed to any of these environmental risk factors at a young age.

The occurrence of SMNs in RB patients is also closely linked to germline variants in the *RB1* tumor-suppressor gene [9]. Although extrinsic factors such as radiotherapy or chemotherapy with alkylating factors have been associated, germline variants in *RB1* increase the risk of SMNs, even in patients who were only enucleated and were not exposed to radiation or chemotherapy [24,25,26,27]. Our patient did not undergo radiotherapy, neither external beam nor brachytherapy, the cumulative dose of carboplatin as an alkylating agent in VCE chemotherapy regimen did not exceed the recommended, and parents deny occupational, lifestyle or environmental risk exposures. Given the lack of evidence of known extrinsic factors involved in the development of the three malignancies and the presence of a germline pathogenic variant in a gene (*RB1*) whose loss of function is a known mechanism of tumorigenesis, we have convincing elements to ensure that the presence of intrinsic factors was the main responsible for the development of the three neoplasms.

Cell cycle deregulation is a mechanism involved in the appearance and development of most malignant tumors [28]. In RB, this deregulation arises in most cases with the biallelic loss of function of the tumor-suppressor gene *RB1*, whose product acts as a negative regulator of the cell cycle, which in its hypophosphorylated state binds to the transcription factor E2F inhibiting the transcription of genes that promote cell cycle progression. Moreover, in the presence of mitogenic signals, RB protein is hyperphosphorylated by cyclin/CDK complexes, causing the protein to dissociate from E2F, allowing progression through G1 into the S phase of the cell cycle [29]. In EWSs, the main one responsible for cell cycle dysregulation is the chimeric oncogene product resulting from the fusion of the *EWSR1* gene and ETS family genes. The oncoprotein promotes cyclin D1 overexpression and modulates cyclin E levels, leading to RB protein hyperphosphorylation without external mitogenic signals [29,30]. Inactivation of the RB protein is caused by variants in the *RB1* gene, although it can occur, and is an infrequent phenomenon in EWSs. We provide evidence of a nonsense germline pathogenic variant in the *RB1* gene and the presence of the *EWRS1/ERG* fusion gene in EWS tumor tissue in our patient. The germline variant in *RB1* alone was likely sufficient for the cell cycle deregulation that triggered tumorigenesis in all three neoplasms. However, in the case of an EWS, its early development and rapid progression may be due to the additive effect of the loss of function of *RB1* and the presence of the *EWSR1/ERG* fusion gene.

To further support our hypothesis that the germline variant in the *RB1* gene as an intrinsic risk factor is sufficient for the development of RB and multiple SMNs even in the absence of extrinsic risk factors, we performed an extensive literature search of the Pubmed database for articles published through August 2024 to identify case reports of patients with RB who developed at least one SMN and EWS being at least one of these SMNs (Table 1). We observed a wide range of subsequent neoplasms in age of onset, from 4 to 20 (mean = 10.7 (standard deviation (SD) 5.17)) years for the first and 9 to 35.8 (mean = 19.8 SD 12.7) years for the second SMN. Most of these patients were treated with radiotherapy, and no germline pathogenic variant in *RB1* was evidenced. Bilateral RB was present in 12 out of the 18 cases [8,31,32,33,34,35,36,37], and no evidence of germinal cytogenetic alterations was reported. However, such alterations probably conferred a higher risk of SMNs regardless of treatment in these patients. The probability of undiagnosed germline cytogenetic alterations is less in the six patients with unilateral RB [33,37,38,39,40]. Although it is not ruled out, it is likely that in these cases, the choice of treatment and environmental factors have had a greater weight in the development of SMNs. Furthermore, the four patients who developed a second SMN had a previous diagnosis of bilateral RB [8,34,36]; this reinforces the idea of germline alterations in *RB1* as an intrinsic risk factor of high relevance in the development of SMNs in these patients. Radiotherapy is known to increase the risk of SMNs [35]; 61.11% of the patients in our review were exposed to some radiotherapy, which is a frequent treatment that increases this risk for patients with RB for SMNs considering the intrinsic risk of SMNs with patients with hereditary RB and the increased risk with this de novo mutation in the *RB1* gene presented in our case.

## 4. Conclusions

In summary, this case expands the clinical and genetic landscape of RB. To the best of our knowledge, it represents the first report of RB with multiple SMNs for which there is evidence of a germline variant in the *RB1* gene. The *RB1* variant identified in the patient is likely a significant driver of tumorigenesis, contributing to the development of both RB and the two SMNs, even without high doses of alkylating agents and radiotherapy. The effect of the variant in the *RB1* gene has been reported as pathogenic in only one patient, diagnosed at one month of age with bilateral retinoblastoma, who, like our patient, had a de novo mode of inheritance; it is unknown whether a second neoplasia was subsequently found or the patient’s clinical picture. However, additional genetic and environmental factors may also play a role. Genetic testing should be considered for identifying germline variants in patients with RB, especially in bilateral cases. Although identifying these variants is sometimes challenging, the presence of a pathogenic germline variant in the *RB1* gene will, in all cases, signify an extremely high risk for the development of SMNs in these patients and will become high-impact evidence for clinical management and long-term follow-up.

## Figures and Tables

**Table 1:** Case reports of patients with RB who developed at least one SMN and EWS.

Sex	Age at Diagnosis RB	Laterality	Familial Case	Therapy	Age at Diagnosis First SMN	Type	Location	Age at Diagnosis Second SMN	Type	Germline Variant	Reference
F	3 m	B	−	En, QT	5 y	Sarcoma	Left ulna	9 y	EWS	c.306T>A (p.Cys102*)	Present case
F	5 m	B		RT	7 y	EWS	Femur	-	-	NI	Kitchin (1974) Case 1 [31]
F	5 m	B		RT	14 y	EWS	Femur	-	-	NI	Kitchin (1974) Case 1 [31]
F	18 m	B	−	En, BT	9 y	EWS	Right ulna	-	-	NI	Schifter (1983) [32]
F	8 m	B	+	QT, RT	12.5 y	EWS	Right ankle	-	-	NI	Helton (1993) Case 1 [33]
M	2 y	U	−	En	20 y	EWS	Left ilium	-	-	NI	Helton (1993) Case 2 [33]
F	1 y	U	−	En	11 y	EWS	Right ankle	-	-	NI	Helton (1993) Case 3 [33]
F	18 m	B	+	En, RT	4 y	EWS	Right distal femur	9 y	Osteosarcoma	NI	Kay (1996) [34]
F	8 m	U	NS	QT, RT	12 y	EWS	Scapula	-	-	NI	Okeda (1997) [38]
F	12 m	B	−	En, RT, QT	20 y	EWS	Left fibula	29 y	Osteosarcoma	NI	Ceha (1998) [35]
M	9 m	B	NS	QT	15 y	EWS	Metatarsal				Mohney (1998) Case 10 [36]
NS	11 m	B	+	En, RT, QT	10 y	Osteosarcoma	Zygomatic bone	-	-	NI	Aerts (2004) Case 2 [37]
NS	22 m	B	−	En, RT, QT	7 y	Osteosarcoma	Left humerus	-	-	NI	Aerts (2004) Case 10 [37]
NS	30 m	U	+	En, RT	11 y	Malignant mesenchymal tumor	Left maxillary sinus	-	-	NI	Aerts (2004) Case 24 [37]
M	9 m	U	NS	En, QT	5 y	EWS	Right fibula	-	-	NI	Mittal (2008) [39]
F	NS	B	NS	RT	18.4 y	EWS	Knee	35.8 y	Osteosarcoma	NI	Marees (2010) Case 7 [8]
F	NS	B	NS	RT	8 y	Soft tissue	Zygoma	16.3 y	Orbit	NI	Marees (2010) Case 11 [8]
M	36 m	U	NS	En, QT	5 y	EWS	Right distal humerus	-	-	NI	Tahasildar (2011) [40]

F = female; M = male; NS = not specified; B = bilateral; U = unilateral; En = enucleation; QT = chemotherapy; RT = radiotherapy; BT = brachytherapy; NI = not investigated; m = months; y = years.

## Data Availability

Data is contained within the article.
